# Can volumetric magnetic resonance imaging evaluations be helpful in the follow-up of cognitive functions in cognitively normal Parkinson’s disease patients?

**DOI:** 10.55730/1300-0144.5838

**Published:** 2024-01-05

**Authors:** Hasan Armağan UYSAL, Duygu HÜNERLİ, Raif ÇAKMUR, Beril DÖNMEZ ÇOLAKOĞLU, Emel ADA, Görsev YENER

**Affiliations:** 1 Department of Neurology, İzmir University of Economics, Medical Point Hospital, İzmir, Turkiye; 2 Department of Neuroscience, Institute of Health Sciences, Dokuz Eylül University, İzmir, Turkiye; 3 Department of Neurology, Faculty of Medicine, Dokuz Eylül University, İzmir, Turkiye; 4 Department of Radiology, Faculty of Medicine, Dokuz Eylül University, İzmir, Turkiye; 5 Faculty of Medicine, İzmir University of Economics, İzmir, Turkiye; 6 İzmir Biomedicine and Genome Center, İzmir, Turkiye

**Keywords:** Parkinson’s disease, volumetric MRI, cognitive function, Wechsler memory scale-revised, trail making test

## Abstract

**Background/aim:**

In this study, besides the evaluation of gray and white matter changes in cognitively normal Parkinson’s disease (PD-CN) patients with volumetric magnetic resonance imaging (MRI) parameters, it was tried to show that some neuropsychological tests may be impaired in PD-CN patients.

**Materials and methods:**

Twenty-six PD-CN patients and 26 healthy elderly (HC) participants were included in the current study. Global cognitive status was assessed using the mini-mental state examination (MMSE), and the Montreal cognitive assessment scale (MoCA). Attention and executive functions were evaluated using the Wechsler memory scale-revised (WMS-R) digit span test and trail making test (TMT) part A and part B, the Stroop test, semantic and phonemic fluency tests, and clock drawing test. Magnetic resonance imaging (MRI) was acquired according to the Alzheimer’s disease neuroimaging initiative (ADNI) protocol.

**Results:**

There were no significant differences among groups regarding age, sex, handedness, and years of education. In the comparison of the PD-CN group and the HC group, there was a statistical decrease in the total animal scores, lexical fluency, TMT part A and TMT part B scores in the PD-CN group. Subcortical gray matter volumes (GMV) were significantly lower in PD-CN patients. The PD-CN group had a significantly reduced total volume of right putamen and left angular gyrus compared to that in the HC group. We observed that putamen and angular gyrus volumes were lower in PD-CN patients. On the other hand, TMT part B may be a useful pretest in detecting the conversion of mild cognitive impairment in PD.

**Conclusion:**

Significant MRI volumetric measurements and neuropsychological test batteries can be helpful in the clinical follow-up in PD-CN patients.

## 1. Introduction

Parkinson’s disease (PD) is the second most common neurodegenerative disease after Alzheimer’s disease and mainly affects the motor system [1]. Besides motor symptoms, nonmotor symptoms such as anosmia, sleep disorders, autonomic findings, pain, depression, anxiety, apathy, and cognitive impairment can occur at any stage of the disease, even before motor symptoms. The causes of cognitive dysfunction in PD are not fully understood, and a rate that is 25% in the early stages of the disease may increase to 80% in the late stages. It has been shown in many studies that there is volume loss in occipital, parietal, and frontal cortices, as well as atrophy in the hippocampus, in PD with cognitive dysfunction [2–3]. There are few studies showing cortical and subcortical tissue volume loss in cognitively normal PD (PD-CN) patients without a diagnosis of cognitive impairment [4–9]. Testing of multiple cognitive areas in neuropsychological evaluation is quite difficult due to the lack of access to trained neuropsychologists and the variation in the educational and cultural levels of the patients [10]. Previous studies have demonstrated that neuropsychological assessments may be impaired in patients with Parkinson’s disease within normal cognitive test scores ranges [11–12].

The aim of this study is to evaluate gray and white matter changes in PD-CN patients with volumetric magnetic resonance imaging (MRI) parameters. In addition, we aimed to highlight that some neuropsychological tests may be impaired in PD-CN patients.

## 2. Methods

### 2.1. Participant selection

Twenty-six PD-CN patients (mean age 65.69 ± 9.20 years; six females, and 20 males) and 26 healthy elderly participants (HC) (mean age 66. 38 ± 6.84 years; eight females, and 18 males) were included in the current study. Patients with PD-CN were recruited from the Movement Disorders Outpatient Clinic in the Department of Neurology at Dokuz Eylül University Hospital. The diagnosis of idiopathic PD was clinically determined based on the UK Parkinson’s Disease Society Brain Bank criteria [13]. The severity of motor symptoms was assessed by the unified Parkinson’s disease rating scale (UPDRS) part III [14] whereas disease severity was examined using the Hoehn and Yahr scale [15].

The inclusion criteria for patients with PD-CN were as follows: (1) a clinical diagnosis of idiopathic PD; (2) controlled motor symptoms with stable dopaminergic treatment; and (3) Hoehn and Yahr stage III or less. The exclusion criteria for PD-CN group were as follows: (1) a clinical diagnosis of PD-mild cognitive impairment [16] and PD-dementia [17], supported by detailed neuropsychological assessments; (2) a history of psychiatric disorders and/or visual hallucinations with the use of medications affecting cognition (e.g., antidepressants, antipsychotics); (3) a history of drug-induced dopamine dysregulation; (4) the presence of and/or a history of vascular lesions, head trauma, seizures, and/or strokes; (5) severe tremors preventing MRI scans; and (6) treatment with deep brain stimulation, jejunal levodopa, and/or subcutaneous apomorphine. Accordingly, one patient was excluded due to severe motion artifacts in MRIs.

An additional 26 healthy elderly participants were recruited from various community sources through bulletin board announcements. The exclusion criteria for the healthy elderly group were as follows: (1) a history or presence of any neurological abnormalities and/or cognitive impairment (mini-mental state examination, MMSE, scoring ≤27), (2) a history of psychiatric disorders, cerebral atrophy, vascular lesions, head trauma, seizures, strokes, alcohol and/or drug abuse/misuse. Participants with depressive symptoms (scoring >14 on the Yesavage geriatric depression scale, GDS [18–19]) were also excluded from all groups.

All PD-CN patients were undergoing the following anti-Parkinsonian treatment at the time of assessments: L-dopa monotherapy (n = 9), dopamine agonist monotherapy (n = 4), MAO-B inhibitor (n = 1), or a combined treatment (n = 12). Levodopa equivalent daily doses (LEDD) were calculated using a standardized formula for all the dopamine replacement therapies that the PD-CN patients were receiving [20]. The neuropsychological and volumetric MRI assessments of the PD-CN patients were conducted during their “on” periods.

All subjects in this study were participants in the prior study by Hünerli-Gündüz et al. [21]. All participants were provided with written informed consent prior to their voluntary participation in the study. The study protocol was approved by the Non-Invasive Research Ethics Board of Dokuz Eylul University with the approval number of 2018-10-38 on April 12, 2018.

### 2.2. Neuropsychological assessment

Neuropsychological performance was evaluated by trained neuropsychologists. Global cognitive status was assessed using the mini-mental state examination (MMSE, [22]) and the Montreal cognitive assessment scale (MoCA, [23]). Attention and executive functions were evaluated using the Wechsler memory scale-revised (WMS-R) digit span test [24], trail making test (TMT) parts A and B [25], the Stroop test [26], semantic and phonemic fluency tests, and clock drawing test [27].

### 2.3. MRI acquisition, preprocessing, and analysis

MRI scans was acquired according to the Alzheimer’s disease neuroimaging initiative (ADNI)^1^ protocol. For each subject, a high-resolution T1-weighted volumetric MRI scan was obtained at the Dokuz Eylül University Neuroradiology Unit, İzmir, Türkiye, using a 1.5 Tesla Philips Achieva system. This included coronal 3D T1-weighted TFE sequences (TR: 9 ms, TE: 4 ms, FOV: 240 mm, matrix: 256, slice thickness: 1 mm, and NSA: 1). Gray matter volume measurements were performed with the CAT12 Toolbox (Computational Anatomy Toolbox)^2^ within the MATLAB-based (Mathworks, Sherborn, MA, USA) SPM12 software^3^.

First, 3D T1-weighted images were converted from DICOM format to NIFTI format. Secondly, the starting points of the images were manually adjusted so that the x, y, z coordinates of the anterior commissure corresponded to the (0, 0, 0) point. This adjustment aligned the MRI images with the Montreal Neurological Institute (MNI) template. Finally, the segmentation process was performed using the parameters recommended in the CAT12 user manual.

As a result of the segmentation process, the 3D T1-weighted images were separated into gray matter, white matter, and cerebrospinal fluid. The CAT12 “Estimate Mean Values inside Region of Interest (ROI)” function was applied using the LPBA40 (LONI Probabilistic Brain Atlas, 101) atlas to obtain mean volume values for different ROIs. Average volume values for each ROI were then extracted separately.

Gray matter volumes (GMV) were also normalized to account for differences in head size among individuals. The normalization process was performed by multiplying each volume value by the volumetric normalization coefficient automatically calculated by SIENAX (an adaptation of SIENA— Structural Image Evaluation, using Normalization, of Atrophy—for crosssectional measurement) [28].

### 2.4. Statistical analysis

SPSS 25.0 (IBM Corporation, Armonk, NY, USA) and MedCalc 14 (MedCalc Software Ltd, Ostend, Belgium) programs were used to analyze the variables. The conformity of the data to a normal distribution was assessed using the Shapiro–Francia test, while the homogeneity of variance was evaluated with the Levene’s test. In the comparison of two independent groups based on quantitative variables, the independent samples t-test was used with the Bootstrap results, while the Mann–Whitney U test was used with the Monte Carlo results. For the comparison of the categorical variables, with each other, the Pearson chi-squared and Fisher’s exact tests were assessed using the Monte Carlo simulation technique. Sensitivity, specificity, positive predictive value, and negative predictive value ratios for the relationship between the classification of the cut-off value were calculated according to the variables and the actual classification. These metrics were analyzed and expressed using receiver operating curve (ROC) analysis. The logistic regression analysis was performed using the Backward method to determine the cause-effect relationship between the categorical dependent variable and the explanatory variables. Quantitative variables were expressed as mean (standard deviation) and median (minimum–maximum) in the tables, while categorical variables were shown as n (%). The variables were analyzed at a 95% confidence level, and a p-value of less than 0.05 was considered significant.

## 3. Results

The demographic, clinical, and neuropsychological characteristics of the patients and healthy controls are presented in Tables 1 and 2. There were no significant differences among the groups regarding age, sex, handedness, and years of education.

### 3.1. Neuropsychological tests of PD-CN

**Figure 1:**
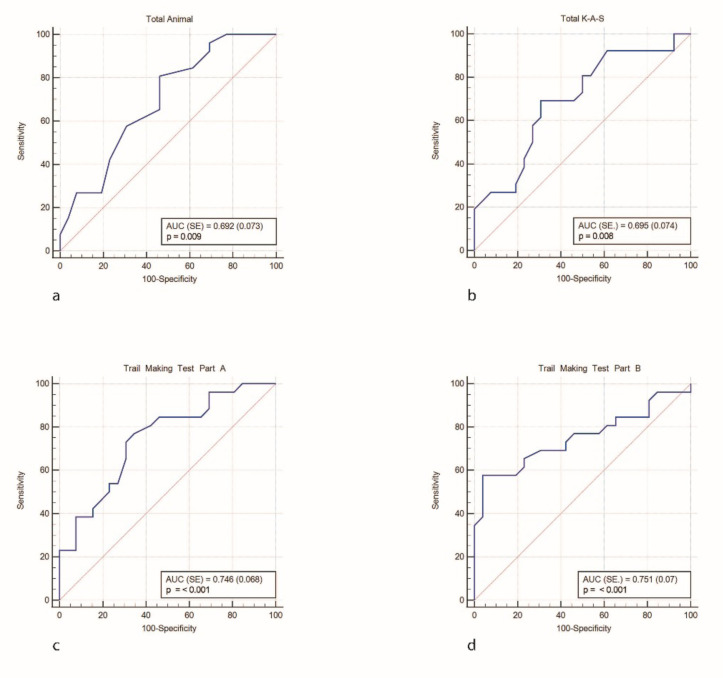
Total animal score (a), K-A-S (b), trail making test part A (c), and part B (d).

In the comparison between the PD-CN group and the HC group, there was a statistically significant decrease in the total animal scores, lexical fluency, TMT part A, and TMT part B scores in the PD-CN group (Table 2). The ROC curves for the neuropsychological test scores were demonstrated in Figure 1. Figure 1a represents the total animal score, Figure 1b indicates the K-A-S score, Figure 1c shows the trail making test part A score, and Figure 1d presents the trail making test part B score.

### 3.2. Volumetry

**Figure 2:**
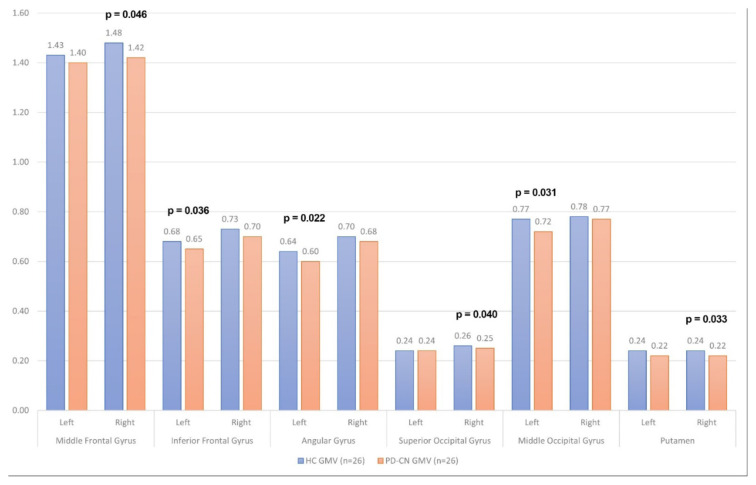
Subcortical GMV volume differences between PD patients and healthy controls.

**Figure 3:**
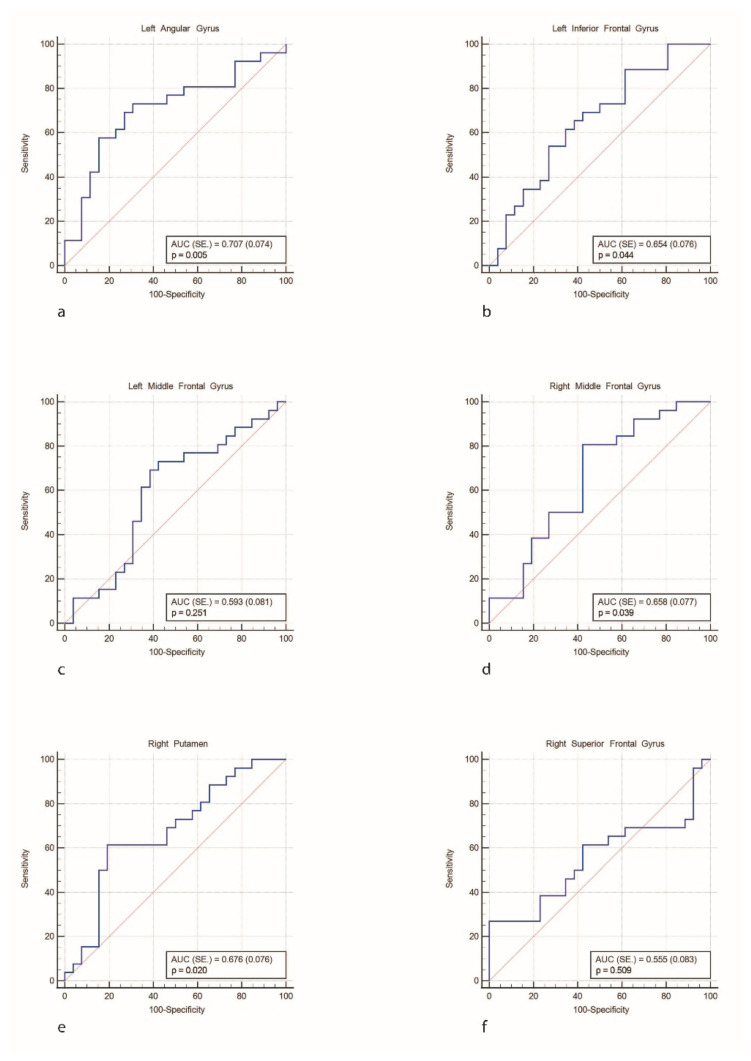
MRI volumetric analysis correlation graphs. Left angular gyrus (a), left inferior frontal gyrus (b), left middle frontal gyrus (c), right middle frontal gyrus (d), right putamen (e), right superior frontal gyrus (f).

There was a statistically significant decrease in the volumes of the right putamen and left angular gyrus in the PD-CN patients compared to healthy controls. Figure 2 demonstrates the subcortical GMV volume differences between PD patients and healthy controls. A comparison of white matter density changes between the PD-CN and HC groups revealed no significant differences (Tables 3 and 4). The ROC curves for MRI volumetric analysis correlation graphs were presented in Figure 3; Figure 3a represents left angular gyrus, Figure 3b the left inferior frontal gyrus, Figure 3c the left middle frontal gyrus, Figure 3d the right middle frontal gyrus, Figure 3e the right putamen, and Figure 3f the right superior frontal gyrus.

### 3.3. Correlation between volumetry and NPT

Regarding the correlations between volumetric analysis and neuropsychological tests in PD-CN, a significant effect was observed between the reduction in putamen and angular gyrus volume and the decline in executive function in PD-CN patients (Table 4).

## 4. Discussion

Cognitive impairments in PD are not limited to a specific cognitive area. Cognitive function declines gradually and heterogeneously in PD, with numerous regions potentially contributing to this decline. There is no standardized neuropsychological test or radiological parameter for the early detection of cognitive dysfunction that accompanies PD. This situation becomes particularly challenging in PD-CN. In the current study, TMT part B, within an extensive neuropsychological test battery, demonstrated differences at the group level in PD-CN. It is noteworthy that commonly employed screening tools, such as MMSE [29,30] and MoCA [31], may remain unimpaired even at the group level during the phase of normal cognition in PD. Since executive functions are among the first to be disrupted in PD, TMT part B may be impaired. Global cognitive scales such as MMSE and MOCA may not reflect the initial impairment in executive function [32–33]. Therefore, TMT part B may be a useful test for detecting cognitive impairment in PD-CN patients.

The volumetric MRI findings of subcortical gray matter in PD-CN patients in the present study indicated a decrease in the volumes of the right putamen and left angular gyrus compared to healthy controls. This finding implies that regional GMV loss occurs in the earliest stages of the disease, even in cognitively intact patients.

The role of subcortical structures in cognition remains elusive. Several recent studies on healthy participants have demonstrated that a higher putamen volume has positive effects on attention and executive functions [3, 34–37]. Previous studies have frequently reported diffuse cortical atrophy in limbic, temporal, prefrontal, occipital, and parietal areas in PD patients with cognitive impairment and dementia [2, 7, 38]. However, information on PD-CN patients is scarce and diverse. Several studies have indicated normal cortical volume in patients with PD-MCI [39–41], while others report dysfunction in temporal, parietal, and occipital cortical involvement patterns [4–9]. The literature shows that GMV loss becomes more prominent in the temporal, parietal, and frontal regions in PD with mild cognitive impairment [1,7,42], and widespread GMV loss occurs as the disease progresses to the dementia phase [36,43–47].

In the present study, we also found that diminished performance on TMT part B test in PD-CN patients was associated with reductions in the volumes of the putamen and angular gyrus, as well as declines in executive function. In the meta-analysis by He et al. [34], structural and functional changes in the brains of PD patients occur at different rates and in different brain regions. Furthermore, increasing gray matter loss as the disease progresses leads to functional deterioration. Atrophy was prominent in the midcingulate gyrus and right supramarginal gyrus in PD-MCI, and in the left insula spreading to the bilateral insular area in PD with dementia.

The pentagon copying test in PD patients without dementia has been shown to be significantly associated with volumetric reductions in cortical regions such as the right complement motor area, left rostral midfrontal cortex, pars triangularis, and left cuneus. This study demonstrated that subtle changes in multiple cognitive domains in PD without dementia are associated with regional volumes in certain systems that play a role in the development of cognitive impairment [9]. Another study showed that both the MMSE and the pentagon copying test reflected regional brain degeneration often found in posterior regions, but that the pentagon copying test was associated with more areas and larger cluster sizes [48]. In a study using TMT B-A scores (the time difference between performance on TMT part A and TMT part B), significant negative correlations were detected bilaterally in the left precentral/middle frontal cortex, right posterior cingulate area, anterior cingulate, and complementary motor area. In addition, it was specifically stated that low GM volume values in these regions may be associated with high TMT B-A time scores [49]. Our data support the use of TMT part B as a tool in patient care to monitor the development of cognitive status in PD-CN patients.

One of the limitations of the current study is the small number of cases and the fact that it was conducted at a single tertiary institution. Another important point is that identifying patients who progress to PD-MCI and determining which neuropsychological test scores decline over time may be crucial. This study will enable a more thorough exploration to establish how certain neuropsychological tests are associated with cortical and subcortical structural alterations as PD-MCI develops. In this study, the demographic variables and clinical characteristics of PD patients were well matched to eliminate the possible confounding effects of age, sex, education, hand dominance, medication use, and disease onset on our results. We suggest that the subcortical volume reductions detected in volumetric MRI can be used as a tool in the follow-up of cognitive functions in PD-CN patients.

## 5. Conclusion

As a remarkable result of our study, we observed that putamen and angular gyrus volumes were lower in PD-CN patients at the group level. On the other hand, TMT part B may be a useful pretest in detecting the conversion of mild cognitive impairment in PD. Therefore, significant MRI volumetric measurements and neuropsychological test batteries can be helpful in the clinical follow-up of PD-CN patients.

## Figures and Tables

**Table 1:** The demographic and clinical characteristics of PD patients and healthy controls.

	Total (n = 52) Mean ± SD	HC-GMV (n = 26) Mean ± SD	PD-CN GMV (n = 26) Mean ± SD	p
**Age**	66.04 ± 8.03	66.38 ± 6.84	65.69 ± 9.20	0.741
**MMSE**	28.79 ± 1.33	29.12 ± 1.11	28.46 ± 1.48	0.077
	**n (%)**	**n (%)**	**n (%)**	
**Sex**				0.755
Female	14 (26.9)	8 (30.8)	6 (23.1)	
Male	38 (73.1)	18 (69.2)	20 (76.9)	
**Education (years)**	11 (5–17)	11 (5–17)	8 (5–15)	**0.049** *
**Hand dominance**				0.49
Left	2 (3.8)	2 (7.7)	0 (0.0)	
Right	50 (96.2)	24 (92.3)	26 (100.0)	
**PD medications**				
Levodopa	9 (34.6)	-	9 (34.6)	-
Dopamine agonist	4 (15.4)	-	4 (15.4)	-
MAO-B inhibitors	1 (3.8)	-	1 (3.8)	-
Combined	12 (46.2)	-	12 (46.2)	-
	**Median (min–max)**	-	**Median (min–max)**	
**Hoehn and Yahr score**	2 (1–3)	-	2 (1–3)	
**UPDRS motor score**	22.5 (6–36)	-	22.5 (6–36)	
**MOCA score**	24.5 (13–30)	-	24.5 (13–30)	
**Disease onset (years)**	3 (1–10)	-	3 (1–10)	-
**Daily levodopa dose**	550 (120–1382)	-	550 (120–1382)	-

HC, healthy elderly participants (control); PD, Parkinson’s disease; GMV, gray matter volume; SD, standard deviation; MMSE, mini-mental state examination; MoCA, Montreal cognitive assessment; min, minimum; max, maximum; n, number, %, percent;

* p < 0.05.

**Table 2:** The neuropsychological test scores of PD patients and healthy controls.

	Total (n = 52) Mean ± SD	HC-GMV (n = 26) Mean ± SD	PD-CN GMV (n = 26) Mean ± SD	p
**Volumes**				
**GM**	41.75 ± 2.47	42.24 ± 2.26	41.27 ± 2.61	0.144
**WM**	36.22 ± 2.27	35.96 ± 2.36	36.49 ± 2.18	0.400
**Matter**				
**Gray**	41.75 ± 2.47	42.24 ± 2.26	41.27 ± 2.61	0.144
**White**	36.22 ± 2.27	35.96 ± 2.36	36.49 ± 2.18	0.400
**Neuropsychological test scores**				
Interference (in seconds)	43.31 ± 13.75	40.81 ± 12.49	45.81 ± 14.72	0.210
Total animal	22.33 ± 4.44	23.92 ± 4.65	20.73 ± 3.64	0.013
Total K-A-S	36.06 ± 12.33	40.23 ± 11.87	31.88 ± 11.54	0.014
	Median (min–max)	Median (min–max)	Median (min–max)	
Total GDS	5 (0–11)	3.5 (0–11)	6 (2–11)	0.065
Digit span forward	6 (4–8)	5.5 (4–8)	6 (4–8)	0.882
Digit span backward	4 (3–7)	4 (3–7)	4 (3–6)	0.420
Total clock drawing	10 (6–10)	10 (8–10)	10 (6–10)	0.112
**Trail making test (measured in the seconds to complete the task)**				
Part A	60.31 ± 23.71	50.42 ±14.31	70.19 ± 27.18	0.010*
Part B	138.27 ± 58.58	112.23 ± 31.91	164.31 ± 67.61	0.004*
Part B-A	78.62 ± 39.81	61.85 ± 25.01	95.38 ± 44.97	0.004*

HC, healthy elderly participants (control); PD, Parkinson’s disease; GMV, gray matter volume; SD, standard deviation; min, minimum; max, maximum; n, number, %, percent; GDS, geriatric depression scale;

* p < 0.05;

** p < 0.001

**Table 3:** The subcortical GMV volumes assessment of the patients and healthy controls.

	Total (n = 52) Mean ± SD	HC GMV (n = 26) Mean ± SD	PD-CN GMV (n = 26) Mean ± SD	p
**Total brain**	77.98 ± 3.35	78.20 ± 3.03	77.75 ± 3.69	0.635
**Both side cerebellar lobe**	5.84 ± 0.60	5.81 ± 0.64	5.86 ± 0.57	0.803
**Both side brainstem**	0.13 ± 0.02	0.13 ± 0.02	0.13 ± 0.02	0.939
**Superior frontal gyrus**				
Left	1.91 ± 0.13	1.93 ± 0.10	1.89 ± 0.14	0.286
Right	1.88 ± 0.13	1.89 ± 0.11	1.86 ± 0.15	0.432
**Middle frontal gyrus**				
Left	1.41 ± 0.11	1.43 ± 0.11	1.40 ± 0.10	0.325
Right	1.45 ± 0.12	1.48 ± 0.13	1.42 ± 0.10	**0.046**
**Inferior frontal gyrus**				
Left	0.66 ± 0.06	0.68 ± 0.07	0.65 ± 0.06	**0.036**
Right	0.71 ± 0.06	0.73 ± 0.06	0.70 ± 0.06	0.149
**Precentral gyrus**				
Left	0.74 ± 0.08	0.75 ± 0.08	0.73 ± 0.08	0.49
Right	0.72 ± 0.07	0.72 ± 0.07	0.73 ± 0.06	0.79
**Middle orbitofrontal gyrus**				
Left	0.34 ± 0.03	0.35 ± 0.03	0.34 ± 0.04	0.166
Right	0.35 ± 0.03	0.36 ± 0.03	0.35 ± 0.03	0.116
**Lateral orbitofrontal gyrus**				
Left	0.23 ± 0.02	0.23 ± 0.03	0.23 ± 0.02	0.350
Right	0.20 ± 0.02	0.20 ± 0.02	0.20 ± 0.02	0.753
**Gyrus rectus**				
Left	0.13 ± 0.01	0.13 ± 0.01	0.13 ± 0.01	0.831
Right	0.13 ± 0.01	0.13 ± 0.01	0.13 ± 0.01	0.623
**Postcentral gyrus**				
Left	0.61 ± 0.07	0.61 ± 0.07	0.60 ± 0.06	0.682
Right	0.58 ± 0.06	0.58 ± 0.06	0.58 ± 0.06	0.712
**Superior parietal gyrus**				
Left	0.76 ± 0.07	0.76 ± 0.06	0.76 ± 0.07	0.790
Right	0.75 ± 0.07	0.77 ± 0.07	0.74 ± 0.07	0.216
**Supramarginal gyrus**				
Left	0.48 ± 0.04	0.49 ± 0.04	0.48 ± 0.05	0.314
Right	0.48 ± 0.04	0.48 ± 0.04	0.47 ± 0.05	0.478
**Angular gyrus**				
Left	0.62 ± 0.06	0.64 ± 0.05	0.60 ± 0.07	**0.022**
Right	0.69 ± 0.07	0.70 ± 0.06	0.68 ± 0.07	0.152
**Precuneus**				
Left	0.44 ± 0.05	0.45 ± 0.05	0.44 ± 0.04	0.377
Right	0.44 ± 0.05	0.45 ± 0.05	0.44 ± 0.05	0.434
**Superior occipital gyrus**				
Left	0.24 ± 0.03	0.24 ± 0.03	0.24 ± 0.03	0.815
Right	0.26 ± 0.03	0.26 ± 0.03	0.25 ± 0.03	**0.040**
**Middle occipital gyrus**				
Left	0.75 ± 0.08	0.77 ± 0.07	0.72 ± 0.08	0.031*
Right	0.78 ± 0.07	0.78 ± 0.07	0.77 ± 0.07	0.458
**Inferior occipital gyrus**				
Left	0.41 ± 0.04	0.42 ± 0.04	0.40 ± 0.05	0.074
Right	0.42 ± 0.04	0.42 ± 0.04	0.42 ± 0.04	0.603
**Superior temporal gyrus**				
Left	1.07 ± 0.07	1.09 ± 0.08	1.06 ± 0.07	0.119
Right	1.01 ± 0.09	1.03 ± 0.09	1.00 ± 0.08	0.197
**Middle temporal gyrus**				
Left	0.91 ± 0.08	0.93 ± 0.08	0.89 ± 0.07	0.138
Right	0.96 ± 0.08	0.97 ± 0.10	0.95 ± 0.07	0.336
**Inferior temporal gyrus**				
Left	0.86 ± 0.06	0.87 ± 0.06	0.85 ± 0.05	0.206
Right	0.91 ± 0.08	0.92 ± 0.08	0.89 ± 0.07	0.162
**Lingual gyrus**				
Left	0.48 ± 0.05	0.48 ± 0.04	0.47 ± 0.05	0.387
Right	0.49 ± 0.05	0.49 ± 0.04	0.48 ± 0.05	0.176
**Fusiform gyrus**				
Left	0.54 ± 0.04	0.55 ± 0.05	0.54 ± 0.04	0.647
Right	0.53 ± 0.04	0.54 ± 0.04	0.53 ± 0.04	0.489
**Insula**				
Left	0.38 ± 0.04	0.38 ± 0.03	0.37 ± 0.04	0.532
Right	0.36 ± 0.03	0.36 ± 0.03	0.35 ± 0.03	0.581
**Cingulate gyrus**				
Left	0.51 ± 0.04	0.52 ± 0.04	0.50 ± 0.04	0.13
Right	0.58 ± 0.05	0.59 ± 0.04	0.58 ± 0.06	0.321
**Caudate**				
Left	0.16 ± 0.02	0.16 ± 0.02	0.16 ± 0.02	0.651
Right	0.15 ± 0.02	0.15 ± 0.02	0.15 ± 0.02	0.898
**Putamen**				
Left	0.23 ± 0.03	0.24 ± 0.03	0.22 ± 0.03	0.056
Right	0.23 ± 0.03	0.24 ± 0.03	0.22 ± 0.03	0.033*
**Hippocampus**				
Left	0.23 ± 0.02	0.23 ± 0.02	0.23 ± 0.02	0.206
Right	0.24 ± 0.02	0.24 ± 0.02	0.24 ± 0.02	0.252
**Cuneus**				
Left	0.21 ± 0.03	0.22 ± 0.02	0.21 ± 0.03	0.315
Right, median (min–max)	0.23 (0.16–0.26)	0.23 (0.18–0.25)	0.22 (0.16–0.26)	0.107
**Parahippocampal gyrus**				
Left, median (min–max)	0.25 (0.18–0.29)	0.24 (0.21–0.29)	0.25 (0.18–0.28)	0.999
Right	0.25 ± 0.02	0.26 ± 0.02	0.25 ± 0.02	0.317

**Table 4:** Subcortical GMV and TMT part B assessment.

*Dependent reference*	*Age and sex adjusted*				*Age and sex not adjusted*			
*Group: (PD-CN-GMV)*	Odds ratio	95% C.I. for odds ratio		p	Odds ratio	95% C.I. for odds ratio		p

		Lower	Upper			Lower	Upper	
**Trail making test part B (>154)**	94.1	4.7	1882.1	0.003*	75.6	5.7	997.1	0.001**
**Left Angular Gyrus (≤0.61)**	12.7	1.5	111.5	0.022*	9.5	1.5	61.4	0.018*
**Right putamen (≤0.22)**	17.2	1.9	152.7	0.011*	11.0	1.7	69.5	0.011*
	**Cut point**	**PD-CN** **GMV**	**HC** **GMV**	**All**	**Cut point**	**PD-CN** **GMV**	**HC** **GMV**	**All**
**Predicted ratio**	**0.617**	**76.9**	**92.3**	84.6	**0.617**	**80.8**	**92.3**	86.5

## Footnotes

1 Alzheimer’s Disease Neuroimaging Initiative (2016). MRI Acquisition [online]. Website https://www.adni.loni.usc.edu [accessed 30 December 2022].

2 Computational Anatomy Toolbox for SPM (2020). Voxel-based morphometry [online]. Website http://dbm.neuro.uni-jena.de/cat/ [accessed 13 March 2023].

3 Statistical Parametric Mapping (2020). SPM12 software [online]. Website http://www.fil.ion.ucl.ac.uk/spm/software/spm12 [accessed 13 March 2023].
